# Effect of HIV Envelope Vaccination on the Subsequent Antibody Response to HIV Infection

**DOI:** 10.1128/mSphere.00738-19

**Published:** 2020-01-29

**Authors:** Zanele Ditse, Nonhlanhla N. Mkhize, Michael Yin, Michael Keefer, David C. Montefiori, Georgia D. Tomaras, Gavin Churchyard, Kenneth H. Mayer, Shelly Karuna, Cecilia Morgan, Linda-Gail Bekker, Koleka Mlisana, Glenda Gray, Zoe Moodie, Peter Gilbert, Penny L. Moore, Carolyn Williamson, Lynn Morris

**Affiliations:** a National Institute for Communicable Diseases of the National Health Laboratory Service (NHLS), Johannesburg, South Africa; b Department of Virology, University of the Witwatersrand, Johannesburg, South Africa; c Department of Medicine, Columbia University, New York, New York, USA; d Department of Medicine, University of Rochester, Rochester, New York, USA; e Duke Human Vaccine Institute, Duke University Medical Center, Durham, North Carolina, USA; f Aurum Institute, Parktown, South Africa; g School of Public Health, University of Witwatersrand, Johannesburg, South Africa; h Fenway Health, Beth Israel Deaconess Medical Center, Harvard Medical School, Boston, Massachusetts, USA; i Vaccine and Infectious Disease Division, Fred Hutchinson Cancer Research Center, Seattle, Washington, USA; j Institute for Infectious Diseases and Molecular Medicine, University of Cape Town, Cape Town, South Africa; k University of Kwa-Zulu Natal, Durban, South Africa; l National Health Laboratory Service, Johannesburg, South Africa; m South African Medical Research Council, Cape Town, South Africa; n Department of Biostatistics, University of Washington, Seattle, Washington, USA; o Centre for the AIDS Programme of Research in South Africa (CAPRISA), Nelson R. Mandela School of Medicine, University of KwaZulu-Natal, Durban, South Africa; Icahn School of Medicine at Mount Sinai

**Keywords:** HIV vaccines, HIV breakthrough infections, HIV-specific binding antibodies, broadly neutralizing antibodies, DNA/MVA vaccines, rAd5, HIV-1 envelope, binding antibody epitopes

## Abstract

Analysis of breakthrough HIV-1 infections could elucidate whether prior vaccination primes relevant immune responses. Here, we measured HIV-specific antibody responses in 14 South African volunteers who acquired HIV infection after participating in phase 1/2 trials of envelope-containing immunogens. Serum samples were collected annually following HIV-1 infection from participants in trials HVTN 073 (subtype C, DNA/MVA, phase 1 trial, *n* = 1), HVTN 086 (subtype C, DNA/MVA/gp140 protein, phase 1 trial, *n* = 2), and HVTN 204 (multisubtype, DNA/adenovirus serotype 5 [Ad5], phase 2 trial, *n* = 7) and 4 placebo recipients. Binding and neutralizing antibody responses to Env proteins and peptides were determined pre- and post-HIV infection using an enzyme-linked immunosorbent assay and the TZM-bl cell neutralization assay, respectively. HIV-infected South African individuals served as unvaccinated controls. Binding antibodies to gp41, V3, V2, the membrane-proximal external region (MPER), and the CD4 binding site were detected from the first year of HIV-1 subtype C infection, and the levels were similar in vaccinated and placebo recipients. Neutralizing antibody responses against tier 1A viruses were detected in all participants, with the highest titers being to a subtype C virus, MW965.26. No responses were observed just prior to infection, indicating that vaccine-primed HIV-specific antibodies had waned. Sporadic neutralization activity against tier 2 isolates was observed after 2 to 3 years of HIV infection, but these responses were similar in the vaccinated and placebo groups as well as the unvaccinated controls. Our data suggest that prior vaccination with these immunogens did not alter the antibody responses to HIV-1 infection, nor did it accelerate the development of HIV neutralization breadth.

**IMPORTANCE** There is a wealth of information on HIV-specific vaccine-induced immune responses among HIV-uninfected participants; however, data on immune responses among participants who acquire HIV after vaccination are limited. Here we show that HIV-specific binding antibody responses in individuals with breakthrough HIV infections were not affected by prior vaccination with HIV envelope-containing immunogens. We also found that these vectored vaccines did not prime tier 2 virus-neutralizing antibody responses, which are thought to be required for prevention against HIV acquisition, or accelerate the development of neutralization breadth. Although this study is limited, such studies can provide insights into whether vaccine-elicited antibody responses are boosted by HIV infection to acquire broader neutralizing activity, which may help to identify antigens relevant to the design of more effective vaccines.

## Abstract (precis)

There is a wealth of information on HIV-specific vaccine-induced immune responses among HIV-uninfected participants; however, data on immune responses among participants who acquire HIV after vaccination are limited. Here we show that HIV-specific binding antibody responses in individuals with breakthrough HIV infections were not affected by prior vaccination with HIV envelope-containing immunogens. We also found that these vectored vaccines did not prime tier 2 virus-neutralizing antibody responses, which are thought to be required for prevention against HIV acquisition, or accelerate the development of neutralization breadth. Although this study is limited, such studies can provide insights into whether vaccine-elicited antibody responses are boosted by HIV infection to acquire broader neutralizing activity, which may help to identify antigens relevant to the design of more effective vaccines.

## INTRODUCTION

Despite advances in understanding the human immune response to HIV infection, there is no licensed vaccine capable of inducing protective immunity. HIV continues to be a major threat, claiming 1 million lives and causing 1.8 million new infections annually, even in the face of a massive antiretroviral treatment program (1). An HIV vaccine is therefore still urgently needed and could have a major public benefit in reducing the ongoing burden of HIV infection.

Several different vaccination concepts to prevent HIV acquisition have been assessed in human efficacy trials. Monomeric gp120 envelope (Env) proteins, aimed at inducing antibody responses, were first tested in the VAX003 and VAX004 trials but elicited neutralizing antibodies only against tier 1A viruses (which are the easiest to neutralize) (2). These vaccines did not induce broadly neutralizing antibodies (bNAbs) and failed to reduce HIV acquisition or impact the set-point viral load (3–5). In order to better prime immune responses, adenovirus (Ad), vaccinia virus, and canarypox virus vaccine vectors able to express HIV gene products were developed (6). Recombinant adenovirus (rAd) was first tested in the STEP efficacy trial (the HVTN 502 trial), but it also failed to reduce HIV acquisition or the viral load set point postinfection (7–9). To further stimulate T-cell breadth, adenovirus serotype 5 (Ad5) was primed with DNA containing multisubtype HIV envelope genes and tested in the HVTN 505 trial (this vaccine was also tested in the HVTN 204 trial). While this vaccine induced binding antibodies to gp41 and HIV gp120, CD4^+^ T-cell responses to HIV-1 Env, and tier 1A virus-neutralizing antibodies, it, too, failed to provide protection against HIV infection (10, 11).

The combination approach, first tested in the RV144 trial, employed a heterologous prime-boost strategy, in which the viral envelope was a feature both of the vector insert and of the Env gp120 construct. The RV144 trial demonstrated moderate efficacy but no effect on the set-point viral load (12). Antibody responses against the V1V2 region of Env as well as lower levels of Env-specific IgA antibodies were identified as immune correlates of the risk of HIV-1 infection (13). Currently, two vector-Env combinations are undergoing efficacy testing in humans: the HVTN 702 study uses a subtype C version of the RV144 regimen, and the HVTN 705/706 study uses an Ad26 vector with optimized sequence inserts and a gp140 protein boost (www.hvtn.org).

In South Africa, the country with the highest HIV infection burden, researchers have been pursuing vaccines against the dominant subtype in the region: HIV-1 subtype C. HVTN 073/SAAVI 102 and HVTN 086/SAAVI 103 were early-phase trials of immunogens, based on the subtype C TV1.21 strain, developed in South Africa (14, 15). HVTN 073 was the first trial to assess the safety and immunogenicity of this DNA/MVA prime-boost regimen. This regimen induced a high frequency of T-cell immune responses but low levels of binding responses to HIV antigens and no neutralizing responses (15). This DNA/MVA regimen, when boosted with a gp140 protein (also tested in HVTN 086), induced binding and tier 1A virus-neutralizing responses but no broadly neutralizing antibody activity (14).

Although there has been a detailed characterization of the vaccine-induced immune responses among HIV-uninfected participants, there are few data available on the immune responses among participants who acquire HIV after vaccination. A study which assessed the effect of Env gp120 vaccination in the VAX004 trial on the subsequent neutralizing antibody responses to HIV-1 infection found no significant differences between HIV-infected vaccinated and placebo recipients (2). However, these responses were assessed at 1 year postinfection and so did not explore whether preinfection vaccination altered the emergence of neutralization breadth, which takes 2 to 4 years to develop in infected individuals (16–18). In this study, we measured binding and the neutralizing antibody responses among South African participants who, despite being at low risk, became HIV infected following vaccination with Env-containing vaccine regimens over a period of 6 years. Our data from this small study of infected participants in the HVTN 073, HVTN 086, and HVTN 204 trials show that prior vaccination with these regimens has no substantial impact on the antibody response to infection.

## RESULTS

### Demographic and clinical features of study participants.

South African volunteers in phase 1/2 HIV vaccine trials of prime-boost regimens who became HIV infected either during or following the trial were recruited into the HVTN 404 trial (Table 1). A total of 24 HIV-infected participants were identified. Multiple samples were available from 14 of these participants and were included in this study (2 from HVTN 073, 2 from HVTN 086, and 10 from HVTN 204). The majority of participants were female (10/14, 71%), and their median age was 28 years (interquartile range, 20 to 48 years), which was comparable between the 10 vaccine and the 4 placebo recipients (Table 2). The participants were predominantly from Cape Town (9/14, 64%) and Soweto (4/14, 29%) in South Africa, and most (12/14, 86%) received all vaccinations, with half becoming HIV-1 infected while on the parent protocol (Table 2). Samples were collected prior to HIV infection and approximately every 12 months thereafter for up to 6 years post-HIV infection. There were no significant differences in the viral loads between infected vaccine and placebo recipients at any time point (all *P* values were >0.05), regardless of the vaccine regimen (see Fig. S1 in the supplemental material).

**TABLE 1:** HIV-infected participants from 3 different HIV vaccine trials enrolled in HVTN 404

HIV vaccine trial^*a*^	No. of HIV-1- infected participants	Regimen received by participants						
		Prime			Boost			
		Mo 0	Mo 1	Mo 2	Mo 3	Mo 4	Mo 5	Mo 6
HVTN 073^*b*^ (36)	1	DNA	DNA	DNA		MVA	MVA	
	1	Placebo	Placebo	Placebo		Placebo	Placebo	
HVTN 086^*c*^ (184)	1	DNA	DNA		MVA + gp140/MF59			MVA + gp140/MF59
	1	MVA	MVA		gp140/MF59			gp140/MF59
HVTN 204^*d*^ (240)	7	DNA	DNA	DNA				rAd5
	3	Placebo	Placebo	Placebo				Placebo

a Values in parentheses are the total number of participants in each trial.

b A DNA/MVA vaccine that contained plasmids expressing subtype C Gag, RT, Tat, and Nef and an HIV-1 truncated *env* gene from Du151 isolate (15).

c A DNA/MVA regimen assessed in HVTN 073 with the inclusion of a gp140/MF59 protein boost from a subtype C TV1.21 strain (14).

d DNA expressing multisubtype *env* (subtype A strain 92RW020, subtype B strain HXB2/BaL, and subtype C strain 97ZA012) boosted with recombinant adenovirus serotype 5 (rAd5) expressing the same *env* genes and a subtype B Gag-Pol fusion protein (19).

**TABLE 2:** Demographic features of HVTN 404 participants

Characteristic	Placebo (*n* = 4)	Vaccine (*n* = 10)	Total
No. (%) of participants by sex			
Male	2	2	4 (29)
Female	2	8	10 (71)
Median age (yr)	27	28	28
No. (%) of participants by site			
Cape Town	3	6	9 (64)
KOSH^*a*^	1	0	1 (7)
Soweto	0	4	4 (29)
No. (%) of participants by vaccine protocol			
HVTN 073	1	1	2 (14)
HVTN 086	0	2	2 (14)
HVTN 204	3	7	10 (72)
No. (%) of participants who:			
Completed vaccination	3	9	12 (86)
Were infected on parent protocol	3	4	7 (50)
Time (mo) from last vaccination to estimated time of HIV infection	16	19	18

a KOSH stands for Klerksdorp, Orkney, Stilfontein, Haartebeesfontein.

10.1128/mSphere.00738-19.1 FIG S1 Comparison of viral load and CD4 counts between vaccine and placebo recipients over time in the HVTN 404 trial. CD4 counts and viral loads were measured from 0 to 6 years post-HIV infection. The date of HIV infection was estimated as the midpoint between the first known HIV-positive date and last serum-negative date. Vaccine recipients are indicated in red, and placebo recipients are indicated in blue. Download FIG S1, TIF file, 0.9 MB. Copyright © 2020 Ditse et al. 2020 Ditse et al. This content is distributed under the terms of the Creative Commons Attribution 4.0 International license.

### Peak vaccine-induced antibody responses.

**FIG 1:**
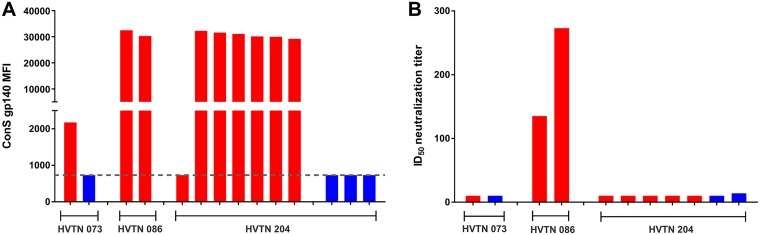
HIV-specific binding and neutralizing antibody responses elicited by the vaccines at peak immunogenicity. (A) Binding antibody responses against HIV-1 ConS gp140 were measured using BAMA for all 10 vaccine recipients (red) and 4 placebo recipients (blue) at 2 to 4 weeks after the last vaccination. The dashed line shows the cutoff for the assay. MFI, mean fluorescence intensity. (B) Historical data for tier 1A virus MW965.26 neutralization at the peak immunogenicity time point for the HVTN 073, HVTN 086, and HVTN 204 trials. Neutralizing antibody data were available for 7 of 10 HVTN 204 participants. Vaccine recipients are indicated in red, and placebo recipients are indicated in blue.

HIV-1 Env binding and neutralizing antibody responses were assessed at the time of peak immunogenicity (2 to 4 weeks after the last vaccination) as part of the parent protocols (14, 15, 19). The DNA/rAd5 regimen used in the HVTN 204 trial induced binding antibodies to HIV-1 ConS (a consensus derived from group M HIV) gp140 in 95% of the participants (19) and in 6 of the 7 vaccine recipients included in this study (Fig. 1A). No neutralizing antibodies to the tier 1A MW965.26 virus were seen in HVTN 204 participants (Fig. 1B; data were available for 5 vaccine recipients). In contrast, both participants in HVTN 086 who received a gp140 protein boost had good responses against MW965.26 as well as ConS gp140 binding (Fig. 1A and B). The single vaccine recipient from the HVTN 073 trial who received the DNA/MVA regimen showed low levels of binding antibodies to ConS gp140 but no tier 1A virus-neutralizing responses (Fig. 1A and B). These data indicate that all 3 vaccine regimens were immunogenic in HIV-uninfected individuals.

### Binding responses to HIV antigens pre- and post-HIV infection.

**FIG 2:**
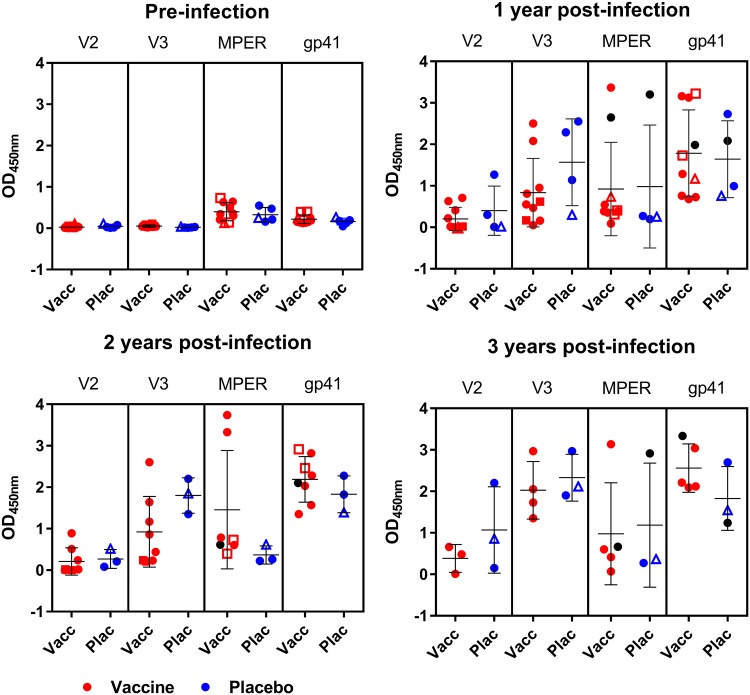
Binding antibody responses to HIV antigens pre- and post-HIV-1 infection. Serum samples from 10 vaccine recipients (Vacc; red) and 4 placebo recipients (Plac; blue) were tested for binding responses against the V2, V3, and MPER peptides and the gp41 ectodomain protein pre-HIV infection and at 1, 2, and 3 years after HIV infection. Open triangles, HVTN 073 participants; open boxes, HVTN 086 participants; closed circles, HVTN 204 participants; black circles, participants who were tested for MPER neutralization responses in the assay whose results are shown in Fig. 4.

Serum samples collected just prior to HIV diagnosis (a median of 18 months postvaccination; Table 2) were assessed for binding to Env peptides and proteins. No binding responses against the V2 and V3 peptides or the gp120 RSC3 proteins (which detect CD4 binding site [CD4bs] antibodies) were observed preinfection, regardless of treatment status or vaccine regimen (Fig. 2). Low levels of binding antibodies against the membrane-proximal external region (MPER) peptide and the gp41 ectodomain were observed in a few participants, including some in the placebo group, and so were unlikely to be vaccine related (Fig. 2).

Following HIV infection, antibodies to V3, MPER, and gp41 were observed in the majority of participants, and their levels remained high for 3 years postinfection (Fig. 2). V2-binding responses developed in fewer participants, and the titers were low in both vaccine and placebo recipients (Fig. 2). Longitudinal data for each participant for each of these 4 antigens over a longer time frame are shown in Fig. S2. Three participants from the HVTN 204 trial had CD4bs responses during the first year of HIV infection, and a fourth developed this specificity at 3 years (Fig. S3). The highest responder was a placebo recipient, whose CD4bs antibodies persisted over 5 years of HIV infection (Fig. S3).

10.1128/mSphere.00738-19.2 FIG S2 Longitudinal analysis of binding antibody response to HIV antigens postvaccination and post-HIV-1 infection. Serum samples from 10 vaccine (red) and 4 placebo (blue) recipients were tested for binding responses against the V2, V3, and MPER peptides and the gp41 ectodomain protein at 0 to 6 years post-HIV infection. Time PI, time since the estimated date of infection. Vaccine recipients are indicated in red, and placebo recipients are indicated in blue. Download FIG S2, TIF file, 1.6 MB. Copyright © 2020 Ditse et al. 2020 Ditse et al. This content is distributed under the terms of the Creative Commons Attribution 4.0 International license.

10.1128/mSphere.00738-19.3 FIG S3 CD4 binding site responses in infected vaccine and placebo recipients over time. Serum samples from 10 vaccine (red) and 4 placebo (blue) recipients were tested for binding responses to the RSC3 wild-type protein and its CD4bs mutant protein by ELISA. The figure shows the fold change in binding responses (OD_450_) of the RSC3 wild-type protein relative to that of the RSC3Δ371IP363N mutant protein. The dotted line indicates a fold change of ≥3, which is considered significant. Vaccine recipients are indicated in red, and placebo recipients are indicated in blue. Download FIG S3, TIF file, 0.5 MB. Copyright © 2020 Ditse et al. 2020 Ditse et al. This content is distributed under the terms of the Creative Commons Attribution 4.0 International license.

### High and persistent gp41 MPER binding responses in HVTN 204 participants postinfection.

**FIG 3:**
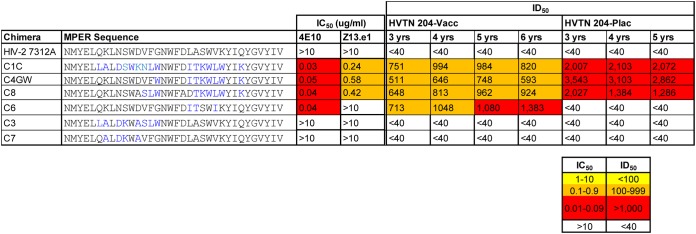
MPER responses in HVTN 204 participants postinfection. Longitudinal serum samples from 2 HVTN 204 participants with MPER binding responses at 3, 4, 5, and 6 years post-HIV infection were tested against the HIV-2/HIV-1 chimeric constructs containing point mutations. Also shown are the sequences carried by the MPER of each engrafted HIV-2/HIV-1 chimeric construct. Amino acid differences between HIV-1 and HIV-2 are indicated in blue. Neutralization titers are shown as ID_50_ and are color coded, as described in the key at the bottom right, with HIV-2 used as a negative control. 4E10 and Z13.e1 were used as positive controls.

Three infected participants from the HVTN 204 trial, one from the vaccine arm and two from the placebo arm, developed gp41 binding antibodies that targeted the MPER and that persisted for 5 to 6 years (Fig. S2). To determine if there were any qualitative differences in these responses, we tested for MPER-neutralizing antibodies using the HIV-2/HIV-1 MPER chimeric constructs (20). High titers against the C1C chimera were seen for the HVTN 204 trial vaccine recipient and one of the HVTN 204 trial placebo recipients, with the latter individual showing 50% inhibitory dose (ID_50_) titers exceeding 2,000 at 3, 4, and 5 years post-HIV infection (Fig. 3). Fine mapping using additional chimeric constructs revealed that the HVTN 204 trial vaccine recipient developed antibodies with a 4E10-like footprint, characterized by potent neutralization of C4GW, C6, and C8. The HVTN 204 trial placebo recipient had a Z13e.1-like footprint, neutralizing C4GW and C8 but not C6 (Fig. 3). These data indicate that both the HVTN 204 trial vaccine and placebo recipients developed neutralizing antibodies targeting the C terminus of MPER, which is not uncommon in HIV-1 subtype C infection (21).

In order to assess the contribution of the MPER-directed antibodies to broad neutralization activity, peptide depletion assays were performed using the MPR.03 peptide. Depletions were done using sera collected at 3, 4, and 5 years postinfection from the 2 participants with MPER responses. Neutralizing activity against C1C as well as 4 heterologous viruses, ConC, Du151, Q23, and CAP45, was tested. A greater than 50% reduction in neutralization activity was observed only for C1C and not for any of the heterologous viruses tested (data not shown). This finding suggests that the MPER responses in these two participants from HVTN 204, one from the vaccine arm and one from the placebo arm, did not contribute to neutralization breadth.

### Tier 1A neutralization responses in vaccine and placebo recipients following HIV infection.

**FIG 4:**
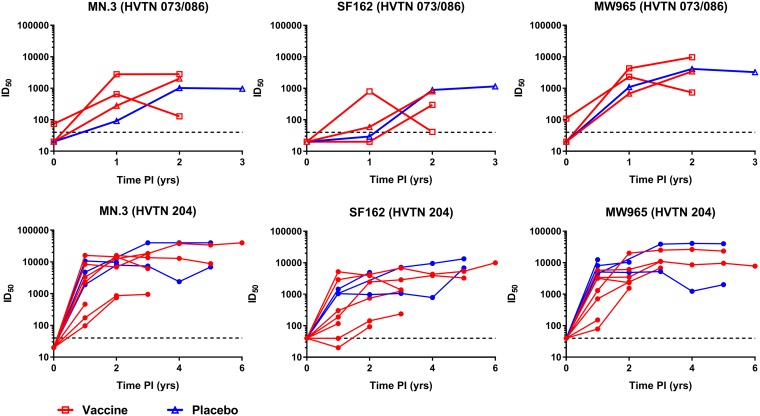
Tier 1A virus-neutralizing responses in infected vaccine and placebo recipients. Serum samples from 10 vaccine (red) and 4 placebo (blue) HIV-infected recipients were assessed for neutralization activity against the MN.3, SF162, and MW965.26 tier 1A viruses. (Top) Data for HVTN 073/086 trial participants; (bottom) data for HVTN 204 trial participants. Time PI, time since the estimated date of infection. Titers of 1:40 are considered positive.

We next assessed neutralization activity against tier 1A isolates MN.3, SF162, and MW965.26 pre- and post-HIV infection. Low neutralizing antibody responses to MN.3 (ID_50_ = 74) and MW965.26 (ID_50_ = 109) were observed for one participant from the HVTN 086 trial prior to HIV infection. For the other participants, no neutralizing responses against tier 1A isolates MN.3, MW965.26, and SF162 were observed prior to infection, consistent with the postvaccination data (Fig. 4).

Following HIV infection, neutralizing antibodies against subtype C isolate MW965.26 followed by subtype B isolates MN.3 and SF162 appeared within the first year and were maintained. There was no statistically significant difference in the titer distributions over time between the vaccine and the placebo recipients for each isolate post-HIV infection (all *P* values were >0.05). Overall, our data suggest that prior vaccination with these *env*-containing regimens had little impact on the antibody response to HIV infection.

### Prior vaccination did not boost tier 2 neutralization responses to HIV infection in HVTN 204.

**FIG 5:**
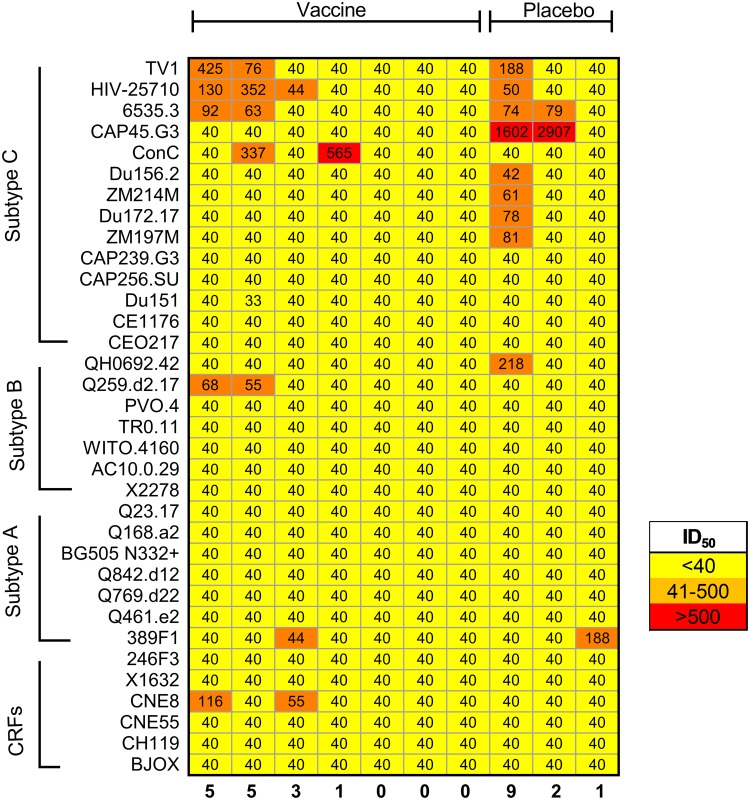
Tier 2 virus-neutralizing responses following HIV infection among vaccine and placebo recipients. Serum samples from 7 vaccine and 3 placebo recipients in the HVTN 204 trial who became HIV infected were assessed for neutralizing activity against 34 viruses from subtypes A, B, and C and circulating recombinant forms (CRFs) at 3 years postinfection. Data are shown as a heatmap, with the titers being color coded as shown in the key at the bottom right. Each column represents one participant, and the total number of viruses neutralized is indicated at the bottom.

Broadly neutralizing antibody responses against 34 tier 2 virus isolates from multiple subtypes were assessed using samples from the infected participants from the HVTN 204 trial (suitable samples were not available for the 3 vaccinees in the other 2 trials). No tier 2 responses were observed for any participant at the preinfection time point, regardless of the vaccine regimen (data not shown). There was sporadic and weak neutralization of the virus panel for some participants at 3 years post-HIV infection, when neutralization breadth is likely to develop. Specifically, 2 vaccinees and 1 placebo recipient neutralized 5 or more members of the virus panel, while others neutralized between 3 and 0 viruses (Fig. 5). Overall, low response rates against tier 2 isolates were observed for both vaccine and placebo recipients, suggesting that prior vaccination did not prime tier 2 virus-neutralizing responses to HIV infection.

### Prior vaccination did not result in higher levels of broadly neutralizing antibodies to HIV infection.

To further understand how the neutralization responses among vaccinated individuals compare to those among unvaccinated individuals, we assessed tier 2 neutralization responses between the HVTN 404 participants and the CAPRISA cohort (17, 22). Since no differences between vaccine and placebo recipients were observed in HVTN 404, the data were pooled for this analysis. The CAPRISA cohort was used as an unvaccinated control group (22), and participants were matched for viral load and CD4 count (Fig. S4). Since the samples from the CAPRISA cohort were previously assessed for their neutralization responses against 18 tier 2 viruses, comparison to HVTN 404 was limited to this smaller panel of shared viruses.

10.1128/mSphere.00738-19.4 FIG S4 Viral load and CD4 count comparison between CAPRISA and HVTN 404 trial participants. The box plots show the viral load and CD4 count comparison between the HVTN 404 and CAPRISA participants at 1, 2, and 3 years post-HIV infection. The date of HIV infection was estimated to be the midpoint between the first known HIV-positive date and last serum-negative date. CAPRISA data are shown in blue, and HVTN 404 data are shown in red. Download FIG S4, TIF file, 0.6 MB. Copyright © 2020 Ditse et al. 2020 Ditse et al. This content is distributed under the terms of the Creative Commons Attribution 4.0 International license.

**FIG 6:**
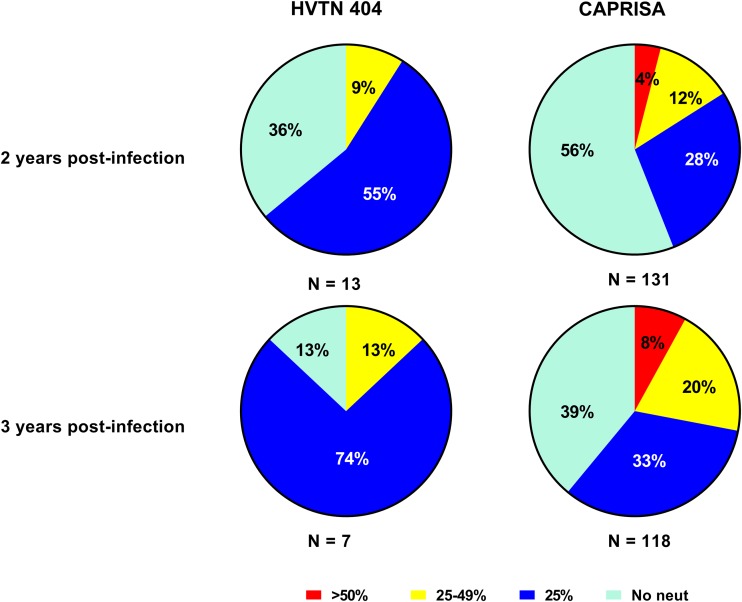
Prior vaccination does not impact the neutralizing antibody responses induced by HIV infection. The pie charts show the tier 2 virus neutralization breadth for HVTN 404 and CAPRISA participants at 2 and 3 years post-HIV infection. A total of 131 CAPRISA participants and 13 HVTN 404 participants were assessed at 2 years postinfection, and 118 CAPRISA participants and 7 HVTN 404 participants were assessed at 3 years postinfection. Neutralization breadth was compared for 18 tier 2 isolates that were also evaluated in the CAPRISA cohort. Red indicates neutralization of >50% of the virus isolates, yellow indicates neutralization of 25 to 49% of the virus panel, indicates neutralization of 25% of the virus panel, and aqua indicates a lack of neutralization breadth (No neut).

A minority of participants in the CAPRISA cohort neutralized >50% of the virus panel, with 5/131 (4%) and 9/118 (8%) showing broadly cross-neutralizing activity at years 2 and 3, respectively (Fig. 6). None of the 13 participants from HVTN 404 tested at year 2 or the 7 participants tested at year 3 showed this level of neutralization, likely due to the lower numbers in HVTN 404. However, a similar percentage of samples from the CAPRISA and HVTN 404 cohorts had 25 to 49% neutralizing activity at both time points. A higher proportion of participants in HVTN 404 showed low-level neutralization of <25%. Overall, our findings suggest that prior vaccination with these envelope-containing regimens did not impact subsequent neutralizing antibody responses upon HIV infection or accelerate the development of neutralization breadth.

## DISCUSSION

Our study is the first to assess the effect of HIV-1 vaccination on subsequent antibody responses in breakthrough infections over the time frame when neutralization breadth is expected to develop. In this small group of individuals, we found that prior vaccination with HIV-1 envelope-containing regimens did not enhance binding or neutralizing antibody responses following HIV-1 infection. A previous study using samples from the VAX004 trial, which assessed a gp120 protein vaccine, demonstrated that prior vaccination had only a minor effect on neutralizing antibody responses following HIV-1 infection (2, 23). Although both these studies are limited, they provide information about whether vaccine-elicited responses are further boosted by HIV infection to acquire broader neutralizing activity. Such information can help to identify relevant antigens and may be used to design more effective vaccines.

All three vaccine regimens included in this study were shown to be immunogenic, as evidenced by the detection of HIV-specific binding antibodies measured at the peak immunogenicity time points (14, 15, 19). Both participants from the HVTN 086 trial showed high levels of binding to ConS gp140 at peak immunogenicity that were equivalent to the levels seen in the participants from HVTN 204. The one participant in the HVTN 204 parent protocol who did not show binding to ConS gp140 was one of the few nonresponders in this trial, which was previously shown to have a high positive response rate (19). Low levels of Env-specific binding antibodies were observed in the single HVTN 073 participant, since the vaccine regimen used in that trial did not include a protein component. However, these primed responses were shown to be significantly boosted following subsequent immunization with a subtype C gp140 protein in the HVTN 073 extension trial (15).

At the preinfection time point (approximately 18 months postvaccination), no significant binding to any of the HIV antigens was observed, confirming that antibody responses to these regimens waned relatively quickly. Indeed, decay kinetic studies in these trials demonstrated a lack of binding antibody responses in all vaccine recipients by 6 months postvaccination (14, 15, 19). However, vaccination is known to elicit durable memory B-cell responses that are boosted on restimulation with the same antigen (24). A similar scenario would be expected in response to infection, assuming that the same antigen is presented by the infecting pathogen, which is the basis of our understanding of how vaccine-mediated protection is functionally effected.

Following HIV infection, we observed Env-specific binding responses to gp41, V3, V2, MPER, and the RSC3 HIV antigens within the first year, and these persisted over time. As the kinetics did not differ between the vaccine and placebo groups, this suggested that HIV infection was the major stimulus. Further support for this comes from the infected participants in the HVTN 204 trial, who developed antibodies to the gp41 ectodomain, which could have been stimulated only by infection, as the vaccine regimen did not contain gp41. Similarly, the HIV Env glycoprotein tested in HVTN 086 contained a V2 deletion, and this may have contributed to the low levels of V2-directed binding responses observed in this study (14). Responses to V3 were similar for all the vaccinated and placebo recipients and across vaccine regimens, all of which contained the V3 region. Overall, our findings suggest that HIV infection did not boost Env-specific binding responses that were primed by vaccination.

All infected participants, irrespective of the vaccine regimen, had high levels of neutralizing responses against tier 1A viruses MW965.26, MN.3, and SF162 that were indistinguishable from those in infected placebo recipients. Although the study of infected participants in VAX004 also suggested no major impact of prior vaccination, the study did find higher titers to the subtype B MN.3 strain. This may be due to the fact that the gp120 protein used in this vaccine was derived from MN.3 and so would be considered an autologous response (4). Sporadic tier 2 virus-neutralizing responses were observed in most of the participants postinfection and these did not differ by vaccination status or regimen. Failure to induce tier 2 virus neutralization responses postvaccination may explain the lack of protection from HIV-1 acquisition in HIV vaccine trials that use these types of vaccines.

Comparison of the infected vaccine recipients from the HVTN 404 trial with the unvaccinated CAPRISA participants suggested that the neutralizing antibody responses primed by vaccination did not differ from the responses that arise following HIV-1 infection. This could suggest that the B-cell lineages that are induced by vaccination do not overlap those that are activated by HIV infection, although this would require an in-depth study of B-cell receptor repertoires. Nevertheless, the inability of these regimens to elicit neutralizing activity highlights the importance of testing newer immunogens that will stimulate B-cell lineages able to mature to acquire broad neutralizing activity, such as germ line-targeting vaccines or trimeric envelope glycoproteins (25).

Limitations of this study include the low numbers of participants available, which likely restricted our ability to detect any significant differences. In addition, the participants received different vaccine regimens, some of which relied on *de novo* Env expression from DNA and from vectored vaccines, likely resulting in lower antigen and antibody levels. Furthermore, a broader range of sequences and antigens as well as more sensitive methods for testing avidity would be required to fully discern if prior vaccination has an impact on antibody responses following HIV infection. Another caveat is that there was a 1-year gap between estimated infection dates and diagnosis dates, which may have affected our measurements. However, it should be noted that samples from vaccinated participants who acquire HIV infection are rare, particularly from phase 1/2 trials that recruits individuals at low risk.

Despite these limitations, our study offers a unique opportunity to examine the antibody responses elicited by different vaccine regimens. In addition, studies of individuals who become infected despite vaccination provide important insights into vaccine antigens that could prime relevant immune responses. Since B cells able to produce bNAbs likely occur at a low frequency, subsequent infection could potentially expand vaccine-primed responses if there is sufficient overlap in the antigenic boost that occurs during infection (26). Our data also suggest that vaccination with Env-containing immunogens did not have a negative impact on the immune response to HIV infection, which is a novel and important observation. Now that larger efficacy trials are under way, additional samples from vaccinated and infected individuals will become available to expand this type of analysis. A thorough understanding of the immune responses elicited by vaccination and infection will play a critical role in the development of an HIV vaccine.

## MATERIALS AND METHODS

### Study population.

Serum samples from 10 vaccine and 4 placebo recipients were collected annually following HIV infection as part of HVTN 404. Participants were recruited from phase 1/2 HIV vaccine trials, conducted in South Africa, in which the vaccine immunogens consisted of HIV-1 *env* inserts and/or Env proteins. These included the HVTN 073/SAAVI 102, HVTN 086/SAAVI 103, and HVTN 204 trials (14, 15, 19). The participants recruited into the parent protocols were assessed as being at low risk of acquiring HIV infection, based on self-reported behavior prior to enrollment. HIV infection was determined by PCR using a Roche Cobas AmpliPrep/Cobas TaqMan HIV-1 qualitative test (version 2.0; Roche Diagnostics, GmbH, Mannheim Germany). Neutralization data from a historic longitudinal cohort of South African individuals enrolled during HIV seroconversion for pathogenesis studies (CAPRISA) were used as data for an unvaccinated control group (17, 20, 22).

### Ethics statement.

The CAPRISA 002 Acute Infection study was reviewed and approved by the research ethics committees of the University of KwaZulu-Natal (E013/04), the University of Cape Town (025/2004), and the University of the Witwatersrand (MM040202). Written informed consent in either English or the local language was obtained from all participants.

### Vaccines.

The HVTN 073/SAAVI 102 trial evaluated the safety and immunogenicity of the DNA/MVA HIV vaccine expressing subtype C Gag, reverse transcriptase (RT), Tat, Nef, and Env from the Du151 isolate (15). HVTN 086/SAAVI 103 evaluated the DNA/MVA regimen with the inclusion of a gp140/MF59 protein boost from a subtype C TV1 strain (14). HVTN 204 tested the immunogenicity of a six-plasmid HIV-1 DNA prime expressing multisubtype Env (from subtype A strain 92RW020, subtype B strain HXB2/BaL, and subtype C strain 97ZA012) boosted by recombinant adenovirus serotype 5 (rAd5) expressing *env* (subtypes A, B, and C) and a subtype B Gag-Pol fusion protein (19). The vaccination schedule for each protocol is shown in Table 1.

### Binding antibody responses.

Binding responses to the ConC V2 linear peptide (CSFNITTELRDKKKKVYALFYRLDIVPLNENSSEYRLINC), the CAP84 V3 linear peptide (TRPNNNTRKSIRIGPGQTFFATNEIIGNIRQAH), the MPR.03 linear peptide (KKKNEQELLELDKWASLWNWFDITNWLWYIRKKK), the HIV-1 gp41 ectodomain protein from the subtype C strain ZA.1197MB, and the resurfaced stabilized gp120 core (RSC3) with its CD4bs mutant, RSC3Δ3711/P363N (27), were assessed by enzyme-linked immunosorbent assays (ELISA). Briefly, 96-well high-binding ELISA plates (Corning, USA) were coated overnight at 4°C with 4 μg/ml of peptide/protein. The plates were washed three times with phosphate-buffered saline containing 0.05% Tween (dilution buffer) and blocked with 5% goat serum, 5% skim milk in dilution buffer for 1 h at 37°C. Serum samples diluted 1:100 were added to the wells, and the plates were incubated for 1 h at 37°C. Unbound antibodies were removed by 4 washes before addition of peroxidase-conjugated goat anti-human IgG (Sigma-Aldrich, St. Louis, MO, USA) at a 1:3,000 dilution. Following incubation with the secondary antibody, the wells were washed four times and developed using 1-Step Ultra tetramethylbenzidine substrate (Thermo Scientific, Waltham, MA, USA). The reaction was stopped with 0.2 M H_2_SO_4_, and the absorbance was read at an optical density at 450 nm (OD_450_) on a microplate reader (Molecular Devices). The results are reported as OD_450_s. The levels of CD4bs antibodies are reported as the fold change in the OD_450_ between CD4bs wild-type and mutant proteins. At the peak immunogenicity time points, the serum IgG binding responses for all 3 study protocols were determined using a binding antibody multiplex assay (BAMA), as described previously (28, 29). Serum samples were serially titrated from a starting dilution of 1:20 or 1:50.

### Neutralization assays.

Neutralization was measured as the reduction in luciferase gene expression following a single round of infection of TZM-bl cells with Env-pseudotyped viruses (30, 31). Sera were tested at a starting dilution of 1:10. Titers were calculated as the 50% inhibitory concentration (IC_50_) or the reciprocal plasma/serum dilution (ID_50_) causing a 50% reduction in the number of relative light units (RLU) compared with that for the virus-treated or untreated control wells. Anti-MPER activity was measured using C1C chimeric HIV-2/HIV-1 MPER constructs containing HIV-1 MPER engrafted into an HIV-2 Env glycoprotein (21). Fine mapping was performed using additional chimeras, C4, C4GW, C6, and C8, which contain point mutations in the MPER. Serum responses with ID_50_s of >1,000 against C1C were further investigated by adsorption of MPER antibodies. Streptavidin-coated magnetic beads (Dynal MyOne streptavidin C1; Invitrogen) were incubated with biotinylated MPER peptides and incubated with serum samples for an hour to deplete MPER-specific antibodies (21). Adsorbed serum samples were tested by ELISA to confirm the depletion of anti-MPER antibodies and in TZM-bl cell neutralization assays to assess the contribution of MPER antibodies to breadth (21).

### Statistical methods.

The Spearman rank correlation was used to assess the correlations between neutralization breadth and viral load or CD4 counts at all time points. All participants were antiretroviral therapy naive at the time of sample collection. A neutralization titer of 1:40 was considered a positive response. Wilcoxon rank-sum tests were used to compare viral loads, CD4 counts, and titers between different groups. *P* values of ≤0.05 were considered statistically significant. All *P* values were two-sided. No adjustments were made for the multiple comparisons for the different endpoints of interest. Statistical analyses were performed using SAS (version 9.4; SAS Institute, Cary, NC, USA) and R statistical (version 3.3.2; R Foundation for Statistical Computing, Vienna, Austria) software.
