# Supplementary figures and images for: Effect of HIV Envelope Vaccination on the Subsequent Antibody Response to HIV Infection
Source: mSphere. 2020 Jan 29;5(1):e00738-19. doi: 10.1128/mSphere.00738-19 (PMC6992371; doi:10.1128/mSphere.00738-19)

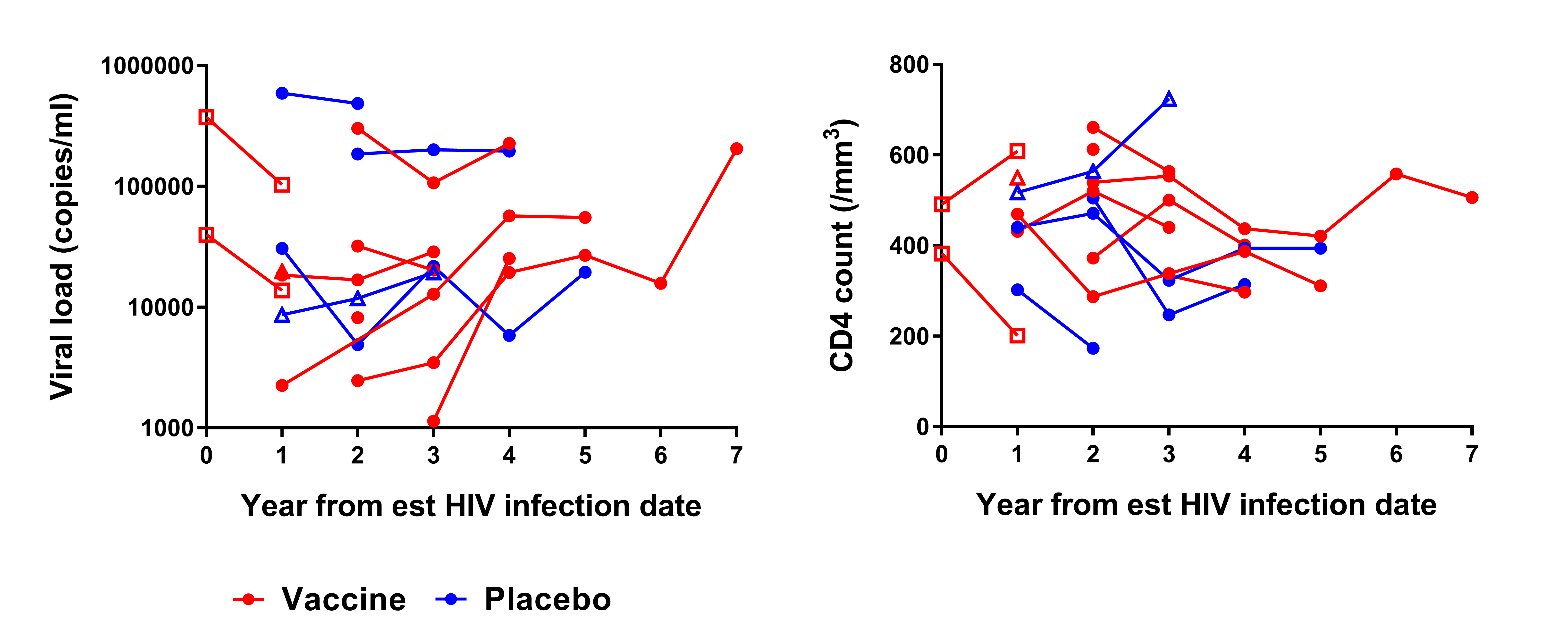

Supplement: FIG S1 [file mSphere.00738-19-sf001.tif]

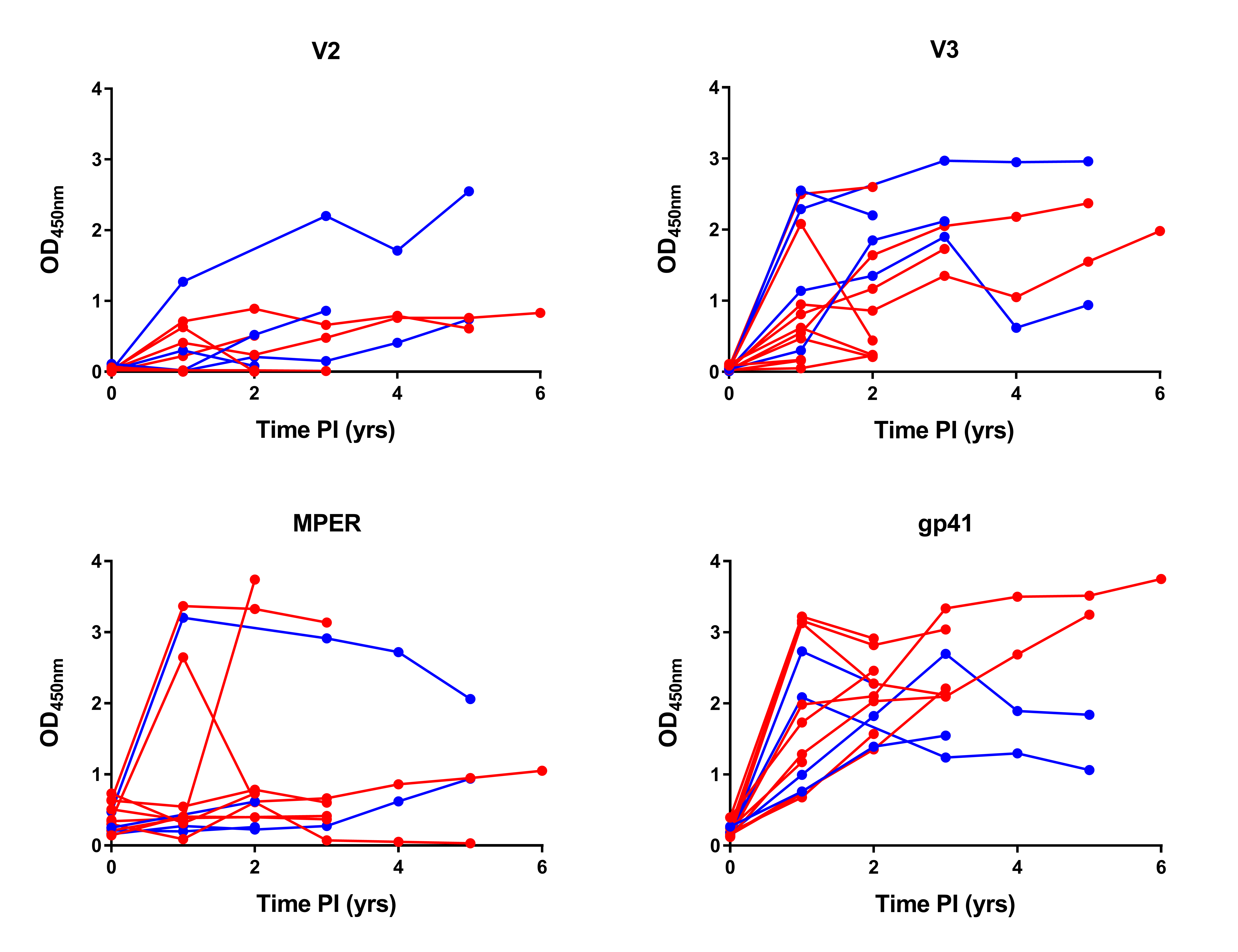

Supplement: FIG S2 [file mSphere.00738-19-sf002.tif]

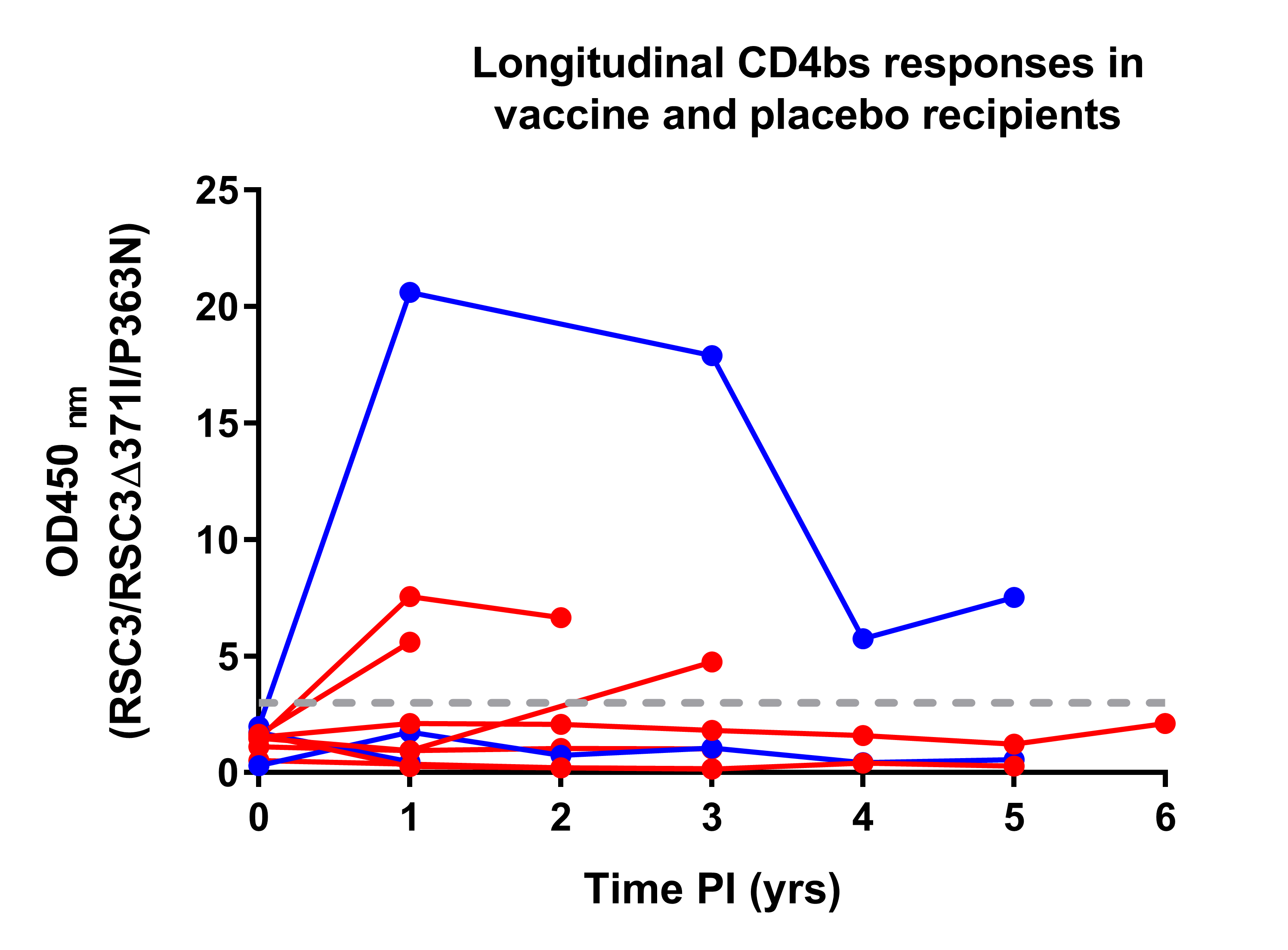

Supplement: FIG S3 [file mSphere.00738-19-sf003.tif]

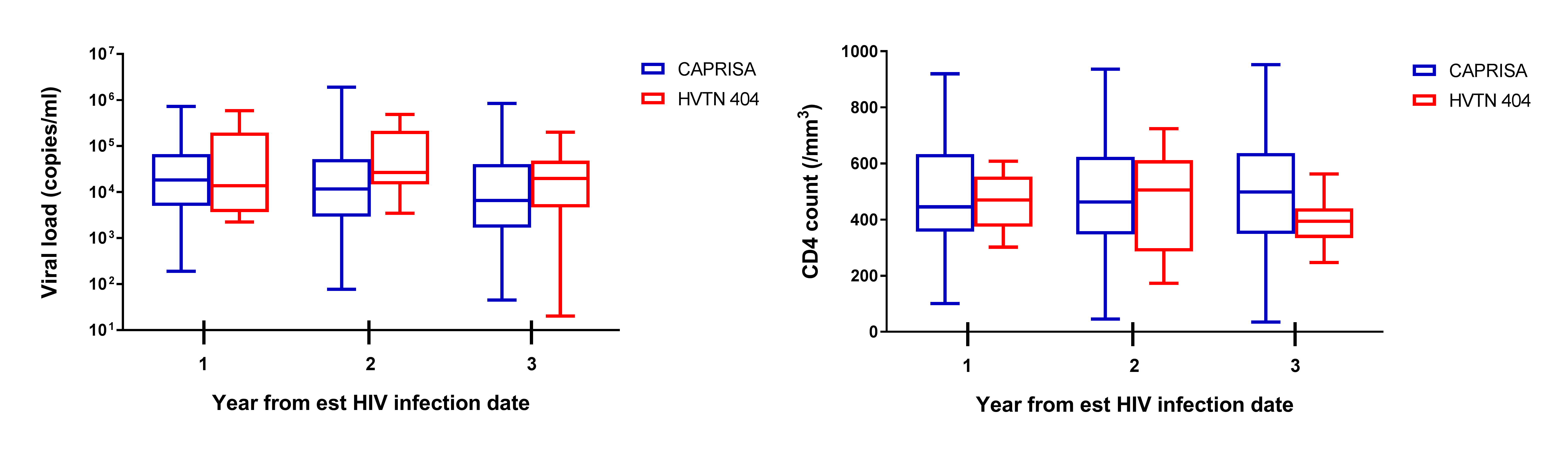

Supplement: FIG S4 [file mSphere.00738-19-sf004.tif]
